# Hyperbaric oxygen therapy modulates immune aging in a personalized manner: a linear mixed-effects analysis

**DOI:** 10.1186/s12979-026-00587-3

**Published:** 2026-08-08

**Authors:** Hai-Tao Chu, Hang Zhao, Shan-Shan Wang, Xin Meng, Di Wang, Xiao-Bo Zhu, Yang Wang, Xue-Wei Yang

**Affiliations:** 1 Department of Hyperbaric Oxygen Medicine, the First Hospital of China Medical University, No. 155 Nanjing North Street, Heping District, Shenyang, 110001 China; 2 The College of Basic Medical Science, Health Sciences Institute, China Medical University, No. 77 Puhe Avenue, Shenbei New District, Shenyang, 110122 China; 3 Canta Medical Tech. Co., Ltd, 2 Puyu Road, Hushitai Sub-District, Shenbei New District, Shenyang, 110122 China; 4 Department of Surgical Oncology and General Surgery, the First Hospital of China Medical University, No. 155 Nanjing North Street, Heping District, Shenyang, 110001 China; 5 Key Laboratory of Precision Diagnosis and Treatment of Gastrointestinal Tumors, Ministry of Education, China Medical University, No. 77 Puhe Avenue, Shenbei New District, Shenyang, 110122 China; 6 Institute of Health Sciences, China Medical University, No. 77 Puhe Avenue, Shenbei New District, Shenyang, 110122 China

**Keywords:** Hyperbaric oxygen therapy, Immunosenescence, Telomere, Cellular senescence, Inflammation, Linear mixed-effects model

## Abstract

**Background:**

Immunosenescence, characterized by telomere shortening, senescence-associated T-cell subsets, and chronic inflammation, is a hallmark of biological aging. Whether these aging-related immune biomarkers can be modulated through short-term intervention and whether responses differ according to individual characteristics remain unclear. This study evaluated the effects of short-term hyperbaric oxygen therapy (HBOT) on senescence-related biomarkers and explored factors associated with individual responsiveness.

**Methods:**

In a prospective cohort study, participants underwent 30 HBOT sessions (2.5 ATA, 100% oxygen, 60 min per session). Relative telomere length (2^-ΔΔCt) and proportions of senescence-associated T-cell phenotypes (CD28⁻/CD27⁻ CD4⁺ or CD8⁺ T cells) were assessed in peripheral blood mononuclear cells (PBMCs), while plasma concentrations (pg/mL) of inflammatory cytokines, including pro-inflammatory cytokines (TNF-α and IL-1β) and the anti-inflammatory cytokine IL-10, were quantified using multiplex bead-based immunoassays at baseline and after 10, 20, and 30 sessions. Linear mixed-effects models were used to evaluate longitudinal HBOT-associated changes and interactions with age, sex, and BMI.

**Results:**

In this clinically heterogeneous cohort comprising apparently healthy individuals and patients receiving HBOT for clinical indications, HBOT was associated with a significant, time-dependent increase in relative telomere length (up to + 29.0% after 30 sessions; *p* < 0.001, Cohen’s d = 0.70) and reduced proportions of senescence-associated T-cell phenotypes, particularly within CD8⁺ T-cell subsets. TNF-α, IL-1β, and IL-10 did not show significant overall time-dependent changes across the cohort. However, subgroup analyses suggested that HBOT-associated responses varied according to age, sex, and BMI: younger (20–39 years) and middle-aged adults (40–59 years), females, and individuals with normal BMI exhibited greater reductions in senescence-associated T-cell phenotypes, whereas middle-aged groups, males showed significant reductions in inflammatory cytokines after 10 sessions.

**Conclusion:**

Short-term HBOT was associated with changes in multiple immunosenescence-related biomarkers, including increased relative telomere length, reduced proportions of senescence-associated T-cell phenotypes, and dynamic modulation of circulating inflammatory profiles. These responses were heterogeneous and influenced by demographic and metabolic factors, suggesting potential differences in individual HBOT responsiveness. These findings indicate that HBOT may modulate selected markers of immune aging; however, further controlled studies are required to determine the durability, clinical relevance, and mechanisms underlying these effects. This study was registered in the Chinese Clinical Trial Registry (ChiCTR2500110103).

**Supplementary Information:**

The online version contains supplementary material available at https://doi.org/10.1186/s12979-026-00587-3.

## Introduction

Aging is a systemic and progressive biological process characterized by functional decline across multiple physiological systems. Among these systems, the immune system undergoes early and clinically relevant alterations, collectively referred to as immunosenescence. This progressive dysfunction not only impairs immune surveillance and vaccine responsiveness, but also contributes to a higher risk of age-related diseases [20, 41]. At the cellular and molecular levels, immunosenescence involves multiple interconnected processes, including telomere attrition, accumulation of senescence-associated immune cell phenotypes, and chronic low-grade inflammation [9, 34, 40].

Telomere attrition is a key molecular feature of cellular aging, particularly in highly proliferative immune cells. In peripheral blood mononuclear cells (PBMCs), telomere length reflects replicative history and cumulative antigenic exposure, and has been associated with immune functional decline in older adults [15]. Therefore, PBMC telomere length may serve as a measurable biomarker of biological aging and immune aging, providing a useful metric for evaluating interventions targeting immune aging-related processes [44].

Senescence-associated T-cell phenotypes represent an important cellular feature of immunosenescence. These cells are characterized by impaired proliferative capacity, altered effector functions, shortened telomeres, and increased inflammatory activity, thereby contributing to age-related immune dysfunction [13, 34, 61]. Phenotypically, T-cell senescence is commonly associated with the loss of co-stimulatory receptors such as CD28 and CD27, particularly within CD4⁺ and CD8⁺ T-cell compartments [5, 27, 38]. CD28⁻ T cells have been widely used as markers of immunosenescence [29], whereas CD27 loss reflects further differentiation toward late-stage effector or effector-memory phenotypes [28, 55].

Inflammaging, a hallmark of immunosenescence, refers to a chronic, low-grade systemic inflammatory state marked by increased circulating inflammatory mediators such as IL-6, TNF-α, and CRP, even without overt infection [17, 20]. Senescent immune cells, through their senescence-associated secretory phenotype (SASP), are now recognized as important contributors to this inflammatory milieu, which may further promote telomere attrition and immune cell senescence, therebyreinforcing immunosenescence [8, 39].

Hyperbaric oxygen therapy (HBOT), which involves intermittent exposure to 100% oxygen under elevated atmospheric pressure, has been increasingly investigated as a potential modulator of aging-related biological pathways [24, 32, 51]. Recent studies suggest that HBOT may influence key aging-related processes, including telomere dynamics, cellular senescence, and inflammatory regulation [6, 19]. In a small clinical trial, HBOT increased telomere length in peripheral blood mononuclear cells (PBMCs) and reduced senescent CD28⁻ T-cell subsets in healthy older adults [25]. Additionally, HBOT has been reported to exert anti-inflammatory effects, potentially through suppression of NF-κB signaling and SASP [23, 58].

However, most existing human studies have been limited by small sample sizes, relatively long intervention protocols, or isolated biological endpoints. Few have simultaneously examined telomere length, senescence-associated T-cell phenotypes, and inflammatory cytokines within an integrated immunosenescence framework, particularly during the early phase of HBOT exposure. Moreover, the potential influence of individual demographic and metabolic factors, such as age, sex, and BMI, on immunological responses to HBOT remains insufficiently explored. These host-related factors may affect the magnitude, direction, and temporal pattern of HBOT-associated biomarker changes, contributing to inter-individual heterogeneity in treatment response. To address these gaps, we conducted a short-term, multi-timepoint HBOT study in a prospective human cohort of 100 participants aged 20–79 years. The primary objective was to longitudinally evaluate HBOT-associated changes in PBMC telomere length, senescence-associated CD4⁺ or CD8⁺ T-cell subsets, including CD28⁻ and CD27⁻ subsets, and inflammatory cytokines after 10, 20, and 30 HBOT sessions. The secondary objective was to investigate whether HBOT-associated immune aging-related biomarker responses varied according to age, sex, and BMI.

## Materials and methods

### Research design

This prospective observational cohort study used a within-subject repeated-measures design to evaluate short-term longitudinal changes in senescence-related biomarkers during a 30-session HBOT protocol. Participants were assessed at four standardized time points: baseline before HBOT and after 10, 20, and 30 HBOT sessions. Peripheral blood samples were collected at each time point to measure relative telomere length in peripheral blood mononuclear cells (PBMCs), senescence-associated T-cell subsets, and inflammatory cytokines. Senescence-associated T-cell subsets were phenotypically defined as CD28⁻ or CD27⁻ populations within CD4⁺ and CD8⁺ T-cell compartments, including CD28⁻CD8⁺, CD27⁻CD8⁺, CD28⁻CD4⁺, and CD27⁻CD4⁺ subsets. Inflammatory cytokines included TNF-α, IL-1β, and IL-10. The study further examined whether age, sex, and BMI modified HBOT-associated longitudinal biomarker trajectories.

A within-subject repeated-measures design was used to capture dynamic changes over the intervention period. Linear mixed-effects models (LMMs) were applied to assess temporal changes across HBOT sessions, with time as the main fixed effect and participant-specific random intercepts used to account for within-subject correlation and inter-individual variability. Models were adjusted for baseline biomarker values, age, sex, and BMI where appropriate. Interaction terms, including Time × Sex, Time × Age, and Time × BMI, were examined to explore whether demographic or metabolic factors modified HBOT-associated biomarker trajectories. When interaction or subgroup effects were of interest, exploratory stratified analyses were further conducted by sex, age group, and BMI category.

### Participants

A total of 100 adult participants were recruited from the First Hospital of China Medical University between 2021 and 2023 (Table 1). The study population consisted of adults receiving hyperbaric oxygen therapy (HBOT), including both patients with clinical HBOT indications and healthy or apparently healthy individuals undergoing HBOT for health management purposes. At enrollment, the clinical status of each participant was recorded. For patients, the primary disease or indication for HBOT was documented. Major comorbidities were also collected from medical records and participant interviews when available. The distribution of clinical status, HBOT indications, and major comorbidities is summarized in Table 1.

**Table 1:** Baseline demographic and clinical characteristics of the study participants

Characteristics	HBOT (*n* = 100)	Senescent analysis (*n* = 93)	Telomere analysis (*n* = 97)
Age			
20-39yrs	14(14.0%)	14 (15.1%)	14 (14.4%)
40-59yrs	42 (42.0%)	40 (43.0%)	41 (42.3%)
60-79yrs	44 (44.0%)	39 (41.9%)	42 (43.3%)
Sex			
Male	39 (39.0%)	38 (40.9%)	39 (40.2%)
Female	61 (61.0%)	55 (59.1%)	58 (59.8%)
BMI			
18.5 ≤ BMI < 24	62 (62.0%)	57 (61.3%)	60 (61.9%)
24 ≤ BMI < 28	38 (38.0%)	36 (38.7%)	37 (38.1%)
Clinical status, n (%)			
Healthy/apparently healthy individuals	27 (27.0%)	26 (28.0%)	27 (27.8%)
Patients with clinical HBOT indications	73 (73.0%)	67 (72.0%)	70 (72.2%)
Primary indication for HBOT, n (%)			
Harmful gas poisoning	2 (2.7%)	2 (3.0%)	2 (2.9%)
Tinnitus or hearing-related disorders	19 (26.1%)	17 (25.4%)	17 (24.2%)
Sleep disturbance/insomnia	10 (13.7%)	10 (14.9%)	10 (14.3%)
Osteomyelitis	23 (31.5%)	21 (31.3%)	22 (31.4%)
Osteonecrosis of the femoral head	11 (15.1%)	10 (14.9%)	11 (15.7%)
Neurological/ischemic conditions	3 (4.1%)	3 (4.5%)	3 (4.3%)
Radiation-induced cystitis/enteritis	2 (2.7%)	2 (3.0%)	2 (2.9%)
Other indications	3 (4.1%)	2 (3.0%)	3 (4.3%)
Major comorbidities, n (%)			
Hypertension	20 (52.6%)	16 (51.6%)	17 (51.5%)
Diabetes mellitus	7 (18.4%)	5 (16.1%)	5 (15.2%)
Cardiovascular disease	2 (5.3%)	1 (3.2%)	2 (6.1%)
Cerebrovascular disease	2 (5.3%)	2 (6.5%)	2 (6.1%)
Malignancy history	4 (10.5%)	4 (12.9%)	4 (12.0%)
Other chronic diseases	3 (7.9%)	3 (9.7%)	3 (9.1%)

Values are presented as n (%). Discrepancies in sample size across analyses are due to missing or poor-quality samples that did not pass laboratory quality control

*HBOT* Hyperbaric oxygen therapy, *BMI* Body mass index

Inclusion criteria were as follows: (1) age between 20 and 79 years; (2) ability to undergo HBOT safely; and (3) willingness to provide peripheral blood samples at all required time points. Exclusion criteria were as follows: (1) history of HBOT within the past 3 months; and (2) contraindications to HBOT, such as pneumothorax, pulmonary bullae, eustachian tube obstruction, or claustrophobia. Written informed consent was obtained from all participants before enrollment.

Participants were stratified by age into three categories: 20–39, 40–59, and 60–79 years, according to widely adopted epidemiological age groupings for adult and elderly health studies [57]. Body mass index (BMI) was categorized using Chinese population-specific criteria: normal weight, 18.5 ≤ BMI < 24 kg/m^2^; overweight, 24 ≤ BMI < 28 kg/m^2^, in accordance with the Working Group on Obesity in China (WGOC) guidelines. The study protocol was approved by the Ethics Review Committee of China Medical University (2021126), and all procedures conformed to the Declaration of Helsinki.

Among the 100 enrolled participants, telomere length data were available for 97 participants, while immunophenotyping data for senescence-associated T-cell subsets were available for 93 participants. The discrepancy in sample size was mainly attributable to incomplete longitudinal sampling and assay-specific data availability, including technical issues during sample processing, quality-control requirements, and incomplete follow-up. Participants with available valid measurements were included in the corresponding analyses.

### Interventions

Participants received HBOT in a Multiplace chamber (YC/Q3224-24, Huaxin, China). The protocol consisted of a 20-min pressurization to 2.5 ATA, during which the participants breathed 100% oxygen via a mask for 60 min (with 10-min air-breaks every 30 min), followed by a 30-min decompression phase. The total treatment course comprised 30 sessions, with a 2-day interval after every 10 sessions.

Throughout the HBOT protocol, participants were monitored for adverse effects, including oxygen toxicity symptoms (e.g., visual disturbances, twitching, seizures), barotrauma (ear/sinus discomfort), and general tolerability. All sessions were supervised by trained hyperbaric medicine personnel, and participants received routine pre- and post-treatment evaluations including vital signs, symptom screening, and pulse oximetry. No pharmacological or behavioral co-interventions were introduced during the HBOT period. Participants were instructed to maintain their usual lifestyle and medication regimen unless otherwise indicated.

### Blood samples

Peripheral blood samples were collected at four time points: pre-HBOT (baseline), and after 10, 20, and 30 HBOT sessions. At each timepoint, approximately 5 mL of venous blood was collected from the antecubital vein using EDTA-coated tubes according to standard procedures. Samples were immediately placed on ice and transported to the laboratory within 30 min.

#### Isolation of peripheral blood mononuclear cells (PBMCs)

Peripheral blood mononuclear cells (PBMCs) were isolated from anticoagulated peripheral blood by density-gradient centrifugation using human peripheral blood lymphocyte separation medium (Ficoll-Paque Plus) according to a standard protocol [21]. Briefly, five milliliters of human peripheral blood lymphocyte separation medium was added to a 15-mL centrifuge tube, and diluted blood (1:1 with 1 × PBS) was carefully layered over the Ficoll solution. The tubes were then centrifuged at 400 × g for 30 min at 20° C.

After centrifugation, the upper plasma layer was removed, and the mononuclear cell layer was carefully collected and washed twice with phosphate-buffered saline. Red blood cell lysis was not performed routinely; PBMCs were separated by density-gradient centrifugation, and the mononuclear cell layer was used for downstream immunophenotyping. Cells were counted using a hemocytometer under light microscopy and resuspended in staining buffer. For each staining tube, 100 μL of cell suspension, corresponding to approximately 1 × 10⁶ cells, was used for antibody staining. Cell viability was not routinely assessed using a dedicated viability assay. Freshly isolated PBMCs were processed promptly after blood collection, and samples with insufficient cell yield or poor flow cytometric acquisition quality were not included in the final immunophenotyping analysis.

#### Telomere length measurement

Relative telomere length was measured in genomic DNA extracted from peripheral blood mononuclear cells using real-time quantitative polymerase chain reaction (RT-qPCR) with SYBR Green PCR Master Mix [7]. Telomere repeat amplification was normalized to the amplification of the single-copy reference gene 36B4 to correct for variations in input DNA amount.

The primer sequences, written in the 5′ to 3′ direction, were as follows:

- telomere-1, GGTTTTTGAGGGTGAGGG TGAGGGTGAGGGTGAGGGT;
- telomere-2, TCCCGACTATCCCTAT CCCTATCCCTATCCCTATCCCTA;
- 36B4u, CAGCAAGTGGGA AGGTGTAATCC; and.
- 36B4d, CCCATTCTATCA TCAACGGGTACAA. The final primer concentrations for the telomere reaction were 270 nM for telomere-1 and 900 nM for telomere-2. The final primer concentrations for the 36B4 single-copy gene reaction were 300 nM for 36B4u and 500 nM for 36B4d. Relative telomere length changes were calculated using the 2^-ΔΔCt method [35], with each participant’s baseline sample used as the internal calibrator.

#### Immunophenotyping of senescent T cells

Flow cytometric immunophenotyping was performed according to standard immunological flow cytometry principles and published guidelines to quantify senescence-associated T-cell subsets defined by CD27 and CD28 expression [11, 47, 59]. CD27 and CD28 were selected because loss of these costimulatory molecules has been associated with repeated antigenic stimulation, late T-cell differentiation, reduced proliferative capacity, and senescence-associated phenotypes. For each staining tube, 100 μL of PBMC suspension, corresponding to approximately 1 × 10⁶ cells, was used for antibody staining. PBMCs were stained with fluorochrome-conjugated mouse anti-human monoclonal antibodies according to the manufacturers’ instructions. The antibodies included FITC-conjugated anti-CD3 (clone UCHT1, BD Biosciences, catalog no. 555916), APC-conjugated anti-CD4 (clone RPA-T4, BD Biosciences, catalog no. 555349), PerCP-Cy™5.5-conjugated anti-CD8 (clone SK1, BD Biosciences, catalog no. 565310), PE-conjugated anti-CD28 (clone CD28.2, BD Biosciences, catalog no. 555729), and PE-conjugated anti-CD27 (clone M-T271, BioLegend, catalog no. 356406). Because CD27 and CD28 antibodies were both PE-conjugated, CD27 and CD28 expression was assessed in separate staining tubes. After staining, cells were washed and resuspended in staining buffer for flow cytometric acquisition. Approximately 20,000 lymphocyte events were acquired per sample using a BD Accuri™ C6 Plus Flow Cytometer (BD Biosciences, San Jose, CA, USA).

The gating strategy was as follows: single cells were identified by forward scatter area (FSC-A) versus forward scatter height (FSC-H) to exclude cell doublets and aggregates; then lymphocyte-enriched cells were identified based on forward scatter and side scatter characteristics; CD3⁺ T cells were then gated from the lymphocyte-enriched population, followed by identification of CD4⁺ or CD8⁺ T-cell subsets; within the CD4⁺ or CD8⁺ T-cell gates, CD28⁻ or CD27⁻ populations were quantified separately; the percentages of CD28⁻CD4⁺, CD27⁻CD4⁺, CD28⁻CD8⁺, and CD27⁻CD8⁺ T-cell subsets were calculated as percentages of their corresponding parent CD4⁺ or CD8⁺ T-cell populations. Samples with insufficient acquired events or poor acquisition quality were excluded from the final immunophenotyping analysis. A representative gating strategy is provided in Figure S1A.

#### Inflammatory cytokine quantification

Circulating inflammatory cytokines were quantified using the RayPlex® Human Inflammation Array Kit (RayBiotech, Inc., Norcross, GA, USA), a multiplex bead-based assay based on flow cytometry (cytometric bead array, CBA), according to the manufacturer’s instructions. The measured cytokines included the pro-inflammatory cytokines tumor necrosis factor-α (TNF-α) and interleukin-1β (IL-1β), as well as the anti-inflammatory cytokine interleukin-10 (IL-10).

Briefly, 25 μL of plasma sample was incubated with fluorescent bead-conjugated capture antibodies in a 96-well filter plate, followed by incubation with biotinylated detection antibodies and streptavidin–phycoerythrin (PE). After washing, samples were analyzed using a BD FACSCalibur™ flow cytometer (BD Biosciences, San Jose, CA, USA), and fluorescence data were processed using RayPlex® data analysis software. Calibration curves were generated using recombinant cytokine standards provided in the kit, and cytokine concentrations were calculated based on median fluorescence intensity (MFI) and expressed as pg/mL.

All samples were measured in duplicate, and intra-assay coefficients of variation (CVs) were maintained below 10%. To minimize inter-assay variation across longitudinal measurements, samples collected from the same participant at different time points were analyzed within the same assay batch.

### Statistical analysis

All statistical analyses were performed using SPSS (version 26.0). Longitudinal changes in relative telomere length (2^-ΔΔCt), senescence-associated T-cell subsets (% within CD4⁺ or CD8⁺ T-cell populations), and cytokine levels (pg/mL) across HBOT sessions were analyzed using linear mixed-effects models (LMMs), which account for within-subject correlations and unbalanced repeated measurements in longitudinal studies [10].

Time (baseline, 10, 20, and 30 sessions) was included as a categorical fixed effect, with subject-specific random intercepts to account for inter-individual variability. Age, sex, BMI, and baseline values were included as fixed-effect covariates. Interaction terms (Time × Age, Time × Sex, and Time × BMI) were tested to evaluate potential differences in treatment trajectories among subgroups.

When significant main or interaction effects were identified, Bonferroni-adjusted pairwise comparisons based on estimated marginal means were performed. Effect sizes were reported as Cohen’s d. Model assumptions were evaluated by examining residual normality and homoscedasticity. Model fit was assessed using Akaike Information Criterion (AIC), Bayesian Information Criterion (BIC), and marginal and conditional R^2^ values.

Missing data were assumed to be missing at random (MAR), and all available observations were included using restricted maximum likelihood (REML) estimation. Cytokine values were log-transformed when required due to non-normal distributions. Statistical significance was defined as a two-sided *p* value < 0.05.

## Results

### HBOT is associated with progressive increases in relative telomere length in human immune cells

Relative telomere length (expressed as fold change relative to baseline, 2^ − ΔΔCt) showed a progressive increase during the HBOT treatment course. The mean value increased from 1.000 at baseline to 1.138 ± 0.049-fold after 10 sessions, 1.202 ± 0.050-fold after 20 sessions, and 1.299 ± 0.056-fold after 30 sessions (Figure S1, Table S7).

Consistent with the observed longitudinal changes, linear mixed-effects modeling adjusted for baseline relative telomere length demonstrated a significant effect of treatment session on relative telomere length (2^-ΔΔCt) (Time effect: F = 11.399, *p* < 0.001). Compared with baseline, relative telomere length increased by 13.9% after 10 sessions (estimate = 0.139, *p* = 0.007, 95% CI [0.039, 0.238], Cohen’s d = 0.33), 20.3% after 20 sessions (estimate = 0.203, *p* < 0.001, 95% CI [0.103, 0.302], Cohen’s d = 0.49), and 29.0% after 30 sessions (estimate = 0.290, *p* < 0.001, 95% CI [0.189, 0.391], Cohen’s d = 0.70) (Table 2).

**Table 2:** Linear mixed-effects model of telomere length changes with main effects of treatment sessions adjusted for baseline levels

Effects	Estimate	SE	Test (*df*)	*p*-value	95% CI	Cohens’ d
Fixed Effects						
Intercept	2.037	0.211	9.679(110.402)	< 0.001	[1.620, 2.455]	
Time(F = 11.399)				< 0.001		
10sessions vs. baseline	0.139	0.051	2.730(304.928)	0.007	[0.039, 0.238]	0.33
20sessions vs. baseline	0.203	0.051	4.003(304.475)	< 0.001	[0.103, 0.302]	0.49
30sessions vs. baseline	0.290	0.051	5.647(306.668)	< 0.001	[0.189, 0.391]	0.70
baseline Δ Ct (F = 25.235)	0.144	0.029	5.023(105.782)	< 0.001	[0.087, 0.201]	
Random Effects						
Subject Intercept Variance	0.040	0.011	Z = 3.798	< 0.001	[0.024, 0.067]	
Residual Variance	0.133	0.011	Z = 12.329	< 0.001	[0.113, 0.155]	
Marginal R2 = 0.144, Conditional R2 = 0.342, AIC = 427.762, BIC = 455.824						

Linear mixed-effects model results estimating the effect of HBOT sessions on telomere length (2^-ΔΔCt). Fixed effects include treatment timepoints (10, 20, and 30 sessions) and baseline telomere expression (ΔCt). Subject ID was modeled as a random intercept

*SE* Standard error, *CI* Confidence interval, *df* Degrees of freedom, *ΔCt* Delta cycle threshold, *AIC* Akaike information criterion, *BIC* Bayesian information criterion

After additionally adjusting for age, sex, and BMI, the time effect remained significant (F = 11.391, *p* < 0.001) (Additional file 1: Table S1). No significant interactions were observed between treatment session and age, sex, or BMI, indicating that the longitudinal trajectory of relative telomere length was not significantly influenced by these demographic characteristics.

### HBOT Modulates senescence-associated T-cell subsets

Flow cytometric immunophenotyping was performed to evaluate longitudinal changes in senescence-associated T-cell phenotypes defined by the loss of CD27 and CD28 expression within CD4⁺ and CD8⁺ T-cell populations. Among the examined subsets, CD8⁺ T-cell populations showed more pronounced longitudinal changes compared with CD4⁺ T-cell populations.

#### Changes in CD28⁻CD8⁺ T-cell proportions during HBOT

The proportion of CD28⁻CD8⁺ T cells showed dynamic changes during HBOT treatment. The mean proportion of CD28⁻CD8⁺ T cells decreased from 48.61 ± 1.88% at baseline to 45.63 ± 1.80% after 10 sessions, followed by 47.13 ± 1.76% and 47.09 ± 1.96% after 20 and 30 sessions, respectively (Figure S1B, Table S8).

Consistent with these observed changes, linear mixed-effects modeling analysis adjusted for baseline values demonstrated a significant effect of HBOT sessions on CD28⁻CD8⁺ T-cell proportions (% of CD8⁺ T cells) (Time effect: F = 3.417, *p* = 0.018) (Table 3). Compared with baseline, CD28⁻CD8⁺ T-cell proportions were significantly reduced after 10 sessions (estimate = −3.180 percentage points, *p* = 0.002, 95% CI [−5.182, −1.178], Cohen’s d = −0.37) and after 30 sessions (estimate = −2.184 percentage points, *p* = 0.039, 95% CI [−4.259, −0.109], Cohen’s d = −0.25). The reduction after 20 sessions showed a similar trend but did not reach statistical significance (estimate = −1.922 percentage points, *p* = 0.062). After additional adjustment for age, sex, and BMI, the time effect remained significant (F = 3.418, *p* = 0.018) (Additional file 1: Table S2).

**Table 3:** Main effects of treatment sessions on senescent T cells: linear mixed-effects model adjusted for baseline levels

T cells	Effects	Estimate	SE	Test (*df*)	*p*-value	95% CI	Cohens’ d
CD28⁻CD8⁺	Fixed Effects						
	Intercept	5.548	2.027	2.706(109.83)	0.008	[1.467, 9.502]	
	Time(F = 3.417)				0.018		
	10sessions vs. baseline	−3.180	1.016	−3.129(255.663)	0.002	[−5.182, −1.178]	−0.37
	20sessions vs. baseline	−1.922	1.023	−1.877(256.325)	0.062	[−3.937, 0.094]	−0.22
	30sessions vs. baseline	−2.184	1.053	−2.073(258.706)	0.039	[−4.259, −0.109]	−0.25
	Baseline(F = 589.172)	0.889	0.366	24.273(91.984)	< 0.001	[0.816, 0.961]	
	Random Effects						
	Subject Intercept Variance	26.715	6.093	Z = 4.384	< 0.001	[17.085, 41.774]	
	Residual Variance	46.699	4.133	Z = 11.299	< 0.001	[39.262, 55.545]	
Marginal R2 = 0.776, Conditional R2 = 0.858, AIC = 2450.178, BIC = 2477.164							
CD27⁻CD8⁺	Fixed Effects						
	Intercept	2.753	1.630	1.689(118.593)	0.094	[−0.474, 5.979]	
	Time(F = 3.306)				0.021		
	10sessions vs. baseline	−2.088	0.932	−2.239(263.628)	0.026	[−3.924, −0.252]	−0.27
	20sessions vs. baseline	−2.824	0.932	−3.029(263.628)	0.003	[−4.660, −0.988]	−0.37
	30sessions vs. baseline	−1.636	0.956	−1.711 (265.988)	0.088	[−3.518, 0.247]	−0.21
	Baseline(F = 812.128)	0.937	0.033	28.498(92.83)	< 0.001	[0.871, 1.001]	
	Random Effects						
	Subject Intercept Variance	20.257	4.611	Z = 4.393	< 0.001	[12.966, 31.648]	
	Residual Variance	39.623	3.459	Z = 11.454	< 0.001	[33.391, 47.018]	
Marginal R2 = 0.846, Conditional R2 = 0.898, AIC = 2434.726, BIC = 2461.850							
CD27⁻CD4⁺	Fixed Effects						
	Intercept	1.350	0.659	2.047(150.507)	0.042	[0.047, 2.653]	
	Time(F = 2.407)				0.068		
	10sessions vs. baseline	−1.089	0.527	−2.064(262.709)	0.040	[−2.127, −0.050]	−0.25
	20sessions vs. baseline	−1.118	0.531	−2.106(263.42)	0.036	[−2.164, −0.073]	−0.26
	30sessions vs. baseline	−0.192	0.543	−0.353(265.515)	0.724	[−1.260, 0.877]	−0.04
	Baseline(F = 1081.869)	0.921	0.028	32.892(90.887)	< 0.001	[0.866, 0.977]	
	Random Effects						
	Subject Intercept Variance	6.202	1.444	Z = 4.294	< 0.001	[3.929, 9.789]	
	Residual Variance	12.765	1.116	Z = 11.441	< 0.001	[10.756, 15.151]	
Marginal R2 = 0.855, Conditional R2 = 0.902, AIC = 2028.393, BIC = 2055.518							
CD28⁻CD4⁺	Fixed Effects						
	Intercept	1.201	0.560	2.145(186.612)	0.033	[0.096,2.306]	
	Time(F = 0.348)				0.791		
	10sessions vs. baseline	−0.386	0.537	−0.719(257.218)	0.473	[−1.443,0.671]	
	20sessions vs. baseline	−0.430	0.540	−0.797(257.910)	0.426	[−1.495,0.634]	
	30sessions vs. baseline	−0.509	0.556	−0.914(260.681)	0.362	[−1.604,0.587]	
	Baseline(F = 802.916)	0.881	0.031	28.336(88.866)	< 0.001	[0.819,0.942]	
	Random Effects						
	Subject Intercept	6.762	1.556	Z = 4.346	< 0.001	[4.307,10.615]	
	Variance						
	Residual Variance	13.068	1.154	Z = 11.321	< 0.001	[10.991,15.539]	
Marginal R2 = 0.777, Conditional R2 = 0.853, AIC = 2006.644, BIC = 2033.65							

Estimated fixed effects from linear mixed model assessing changes in senescent T cell percentages (% CD8 or %CD4) across 10, 20, and 30 HBOT sessions, adjusted for baseline levels

*SE* Standard error, *CI* Confidence interval; *df* Degrees of freedom, *AIC* Akaike information criterion, *BIC* Bayesian information criterion

Further interaction analyses indicated that demographic characteristics modified the longitudinal trajectory of CD28⁻CD8⁺ T-cell changes. A borderline interaction between treatment session and age was observed (Time × Age: F = 2.115, *p* = 0.052) (Table 4). Further pairwise comparisons showed that young adults (20–39 years) exhibited a significantly greater reduction than elderly participants (60–79 years) after 30 sessions (estimate = −7.405 percentage points, *p* = 0.023, 95% CI [−13.758, −1.052]). Age-stratified analyses further demonstrated that young adults showed a progressive reduction in CD28⁻CD8⁺ T-cell proportions, with significant decreases observed after 10 sessions (estimate = −6.277 percentage points, *p* = 0.019) and the largest reduction after 30 sessions (estimate = −11.025 percentage points, *p* < 0.001). In contrast, no significant reductions were observed in the elderly group at any treatment time point (all *p* > 0.05) (Table 5).

**Table 4:** Linear mixed-effects model of senescent T cells including time × age, time × sex and time × Body Mass Index (BMI) Interaction

Effects	Estimate	SE	Test (*df*)	*p*-value	95% CI
CD28⁻CD8⁺					
Time (F = 3.938)				0.009	
Age (F = 4.816)				0.010	
Time × Age (F = 2.115)				0.052	
30sessions × YG (vs. EG)	−7.405	3.226	−2.295 (262.303)	0.023	[−13.758, −1.052]
BMI (F = 2.509)				0.117	
Time × BMI (F = 1.975)				0.118	
20sessions × NW (vs. OW)	−5.020	2.066	−2.430 (257.164)	0.016	[−9.089, −0.951]
Sex (F = 4.535)				0.036	
Time × Sex (F = 2.262)				0.082	
20sessions × M (vs. F)	4.853	2.044	2.374 (257.122)	0.018	[0.828, 8.879]
30sessions × M (vs. F)	4.219	2.114	1.996 (259.974)	0.047	[0.057, 8.381]
CD27⁻CD8⁺					
Time (F = 2.483)				0.061	
Age (F = 1.006)				0.370	
Time × Age (F = 1.320)				0.256	
20sessions × MG (vs. EG)	−4.465	1.990	−2.244 (263.484)	0.026	[−8.383, −0.547]
CD28⁻CD4⁺					
Time (F = 0.176)				0.913	
BMI (F = 0.855)				0.358	
Time × BMI (F = 2.248)				0.083	
20sessions × NW (vs. OW)	−2.809	1.086	−2.588(258.316)	0.010	[−4.947, −0.672]
Sex (F = 3.317)				0.072	
Time × Sex (F = 2.248)				0.083	
20sessions × M (vs. F)	2.751	1.076	2.556 (258.354)	0.011	[0.631, 4.870]
CD27⁻CD4⁺					
Time (F = 2.023)				0.111	
Age (F = 1.838)				0.165	
Time × Age (F = 1.917)				0.078	
20sessions × MG (vs. EG)	−2.812	1.120	−2.511 (263.081)	0.013	[−5.018, −0.607]

Estimated fixed effects from linear mixed model assessing Time × Age, Time × Sex and Time × BMI Interaction in senescent T cells across 10, 20, and 30 HBOT sessions. Time × Sex and Time × BMI Interaction in senescent T cells across 10, 20, and 30 HBOT sessions. YG (young group, 20–39 yrs); MG (middle-aged group, 40–59 yrs); EG (elderly group, 60–79 yrs); F(female); M(male); NW (normal weight, 18.5 ≤ BMI < 24 kg/m2); OW (overweight, 24 ≤ BMI < 28 kg/m2)

*CI* Confidence interval, *df* Degrees of freedom

**Table 5:** Stratified analysis in senescent T cells following hyperbaric oxygen therapy

T cells	Group		10Sessions vs. Baseline	20Sessions vs. Baseline	30Sessions vs. Baseline
CD27⁻CD8⁺	Age	20-39yrs	−2.072 ± 2.409 (0.391)	−2.257 ± 2.409 (0.350)	−0.846 ± 2.403 (0.725)
		40-59yrs	−2.050 ± 1.411 (0.147)	−5.135 ± 1.398 (< 0.001)	−2.849 ± 1.423 (0.046)
		60-79yrs	−2.259 ± 1.404 (0.109)	−0.669 ± 1.417(0.637)	−0.401 ± 1.486 (0.787)
	Sex	male	−0.587 ± 1.437 (0.683)	−1.558 ± 1.437 (0.279)	−0.721 ± 1.507 (0.633)
		female	−3.289 ± 1.218 (0.007)	−3.725 ± 1.218 (0.002)	−2.105 ± 1.233(0.089)
	BMI	18.5 ≤ BMI < 24	−3.207 ± 1.185 (0.007)	−2.627 ± 1.193 (0.028)	−1.451 ± 1.232 (0.240)
		24 ≤ BMI < 28	−0.517 ± 1.487 (0.728)	−3.100 ± 1.473 (0.036)	−1.615 ± 1.501 (0.283)
CD28⁻CD8⁺	Age	20-39yrs	−6.277 ± 2.663 (0.019)	−4.800 ± 2.735 (0.081)	−11.025 ± 2.911 (< 0.001)
		40-59yrs	−3.767 ± 1.538 (0.015)	−1.915 ± 1.538 (0.214)	0.120 ± 1.608 (0.941)
		60-79yrs	−1.627 ± 1.551 (0.295)	−1.005 ± 1.565 (0.521)	−2.188 ± 1.579 (0.167)
	Sex	male	−1.868 ± 1.604 (0.245)	0.909 ± 1.618 (0.575)	−0.047 ± 1.695 (0.978)
		female	−4.191 ± 1.366 (0.002)	−4.036 ± 1.375 (0.004)	−4.039 ± 1.404 (0.004)
	BMI	18.5 ≤ BMI < 24	−4.322 ± 1.343 (0.001)	−3.970 ± 1.351(0.004)	−3.594 ± 1.366(0.009)
		24 ≤ BMI < 28	−1.547 ± 1.649 (0.349)	1.086 ± 1.665 (0.515)	−0.502 ± 1.775 (0.778)
CD27⁻CD4⁺	Age	20-39yrs	−1.994 ± 1.356 (0.143)	0.324 ± 1.393 (0.816)	−0.374 ± 1.352 (0.782)
		40-59yrs	−1.132 ± 0.787 (0.151)	−2.714 ± 0.787 (0.001)	−1.078 ± 0.801 (0.179)
		60-79yrs	−0.744 ± 0.790 (0.348)	0.135 ± 0.797 (0.866)	1.048 ± 0.836 (0.211)
	Sex	male	−0.045 ± 0.814(0.956)	−0.595 ± 0.814 (0.466)	0.224 ± 0.854 (0.793)
		female	−1.846 ± 0.685 (0.008)	−1.434 ± 0.694 (0.040)	−0.335 ± 0.699 (0.632)
	BMI	18.5 ≤ BMI < 24	−1.585 ± 0.671 (0.019)	−0.688 ± 0.680 (0.312)	−0.223 ± 0.698 (0.749)
		24 ≤ BMI < 28	−0.353 ± 0.834 (0.673)	−1.658 ± 0.834 (0.048)	0.074 ± 0.850 (0.931)
CD28⁻CD4⁺	Age	20-39yrs	0.508 ± 1.457 (0.727)	−0.770 ± 1.500 (0.608)	−2.078 ± 1.610 (0.198)
		40-59yrs	−0.693 ± 0.805 (0.390)	−0.344 ± 0.805 (0.669)	0.566 ± 0.843 (0.503)
		60-79yrs	−0.381 ± 0.805 (0.636)	−0.378 ± 0.812 (0.641)	−0.985 ± 0.819 (0.230)
	Sex	male	0.231 ± 0.806(0.775)	1.177 ± 0.813 (0.149)	0.583 ± 0.862(0.499)
		female	−0.855 ± 0.701 (0.223)	−1.610 ± 0.705 (0.023)	−1.175 ± 0.715 (0.101)
	BMI	18.5 ≤ BMI < 24	−0.907 ± 0.688 (0.189)	−1.543 ± 0.693 (0.027)	−0.978 ± 0.701 (0.165)
		24 ≤ BMI < 28	0.362 ± 0.828 (0.662)	1.235 ± 0.836 (0.141)	0.347 ± 0.890 (0.697)

Values represent estimated mean changes (± standard error) in senescent T cells at 10, 20, and 30 HBOT sessions relative to baseline, stratified by sex, age and BMI

*BMI* Body mass index

A similar trend was observed for sex-related differences. Although the Time × Sex interaction did not reach statistical significance (F = 2.262, *p* = 0.082), subgroup comparison indicated that males showed less reductions than females after 20 sessions (estimate = 4.853 percentage points, *p* = 0.018, 95% CI [0.828, 8.879]) and 30 sessions (estimate = 4.219 percentage points, *p* = 0.047, 95% CI [0.057, 8.381]) (Table 4). Sex-stratified analysis demonstrated consistent reductions among females after 10, 20, and 30 sessions (estimate = −4.191, *p* = 0.002; estimate = −4.036, *p* = 0.004; estimate = −4.039, *p* = 0.004, respectively), whereas no significant longitudinal changes were observed in males (Table 5).

BMI-related differences were also observed. Although the Time × BMI interaction was not statistically significant, participants with normal weight (18.5 ≤ BMI < 24) showed greater reductions compared with overweight participants (24 ≤ BMI < 28) after 20 sessions (estimate = −5.020 percentage points, *p* = 0.016, 95% CI [−9.089, −0.951]) (Table 4). BMI-stratified analysis further revealed that the normal BMI group exhibited significant reductions after 10, 20, and 30 sessions (estimate = −4.322, *p* = 0.001; estimate = −3.970, *p* = 0.004; estimate = −3.594, *p* = 0.009, respectively), whereas no significant changes were observed in the overweight group (Table 5).

#### Changes in CD27⁻CD8⁺ T-cell proportions during HBOT

The proportion of CD27⁻CD8⁺ T cells showed dynamic changes during HBOT treatment. Based on flow cytometric measurements, the mean proportion of CD27⁻CD8⁺ T cells decreased from 42.27 ± 1.79% at baseline to 39.84 ± 1.79% after 10 sessions and 39.24 ± 1.84% after 20 sessions, followed by 40.94 ± 1.96% after 30 sessions (Figure S1B, Table S9).

Consistent with these observed changes, linear mixed-effects modeling demonstrated a significant effect of treatment sessions on CD27⁻CD8⁺ T-cell proportions (% of CD8⁺ T cells) (Time effect: F = 3.306, *p* = 0.021) (Table 3). Compared with baseline, CD27⁻CD8⁺ T-cell proportions significantly decreased after 10 sessions (estimate = −2.088 percentage points, *p* = 0.026, 95% CI [−3.924, −0.252], Cohen’s d = −0.27) and 20 sessions (estimate = −2.824 percentage points, *p* = 0.003, 95% CI [−4.660, −0.988], Cohen’s d = −0.37), whereas the reduction after 30 sessions did not reach statistical significance (estimate = −1.636 percentage points, *p* = 0.088). After further adjustment for age, sex, and BMI, the longitudinal effect of treatment sessions remained significant (F = 3.339, *p* = 0.020) (Additional file 1: Table S3).

Further interaction and stratified analyses were performed to explore whether demographic characteristics modified the response of CD27⁻CD8⁺ T-cell proportions. Although the Time × Age interaction did not reach statistical significance, pairwise subgroup comparisons revealed that the middle-aged group (40–59 years) exhibited a greater reduction compared with the elderly group (60–79 years) after 20 sessions (estimate = −4.465 percentage points, *p* = 0.026, 95% CI [−8.383, −0.547]) (Table 4). Age-stratified analysis further demonstrated that the middle-aged group showed the most pronounced reduction after 20 sessions (estimate = −5.135 percentage points, *p* < 0.001) with a sustained reduction after 30 sessions (estimate = −2.849 percentage points, *p* = 0.046), whereas elderly participants did not show significant longitudinal changes (Table 5).

Sex-stratified analysis demonstrated that females exhibited significant reductions in CD27⁻CD8⁺ T-cell proportions after 10 sessions (estimate = −3.289 percentage points, *p* = 0.007) and 20 sessions (estimate = −3.725 percentage points, *p* = 0.002), with a similar but non-significant trend after 30 sessions (estimate = −2.105 percentage points, *p* = 0.089). In contrast, no significant changes were observed among males at any treatment time point.

BMI-stratified analysis indicated differential response patterns. Participants with normal BMI (18.5 ≤ BMI < 24) showed earlier reductions after 10 sessions (estimate = −3.207 percentage points, *p* = 0.007) and 20 sessions (estimate = −2.627 percentage points, *p* = 0.028). In comparison, overweight participants (24 ≤ BMI < 28) demonstrated a delayed response, with a significant reduction observed only after 20 sessions (estimate = −3.100 percentage points, *p* = 0.036).

#### Changes in CD27⁻CD4⁺ T-Cell Proportions During HBOT

The proportion of CD27⁻CD4⁺ T cells showed modest dynamic changes during HBOT treatment. Based on flow cytometric measurements, the mean proportion of CD27⁻CD4⁺ T cells decreased from 17.48 ± 1.21% at baseline to 15.87 ± 1.11% after 10 sessions and 15.99 ± 1.20% after 20 sessions, followed by 17.15 ± 1.30% after 30 sessions (Figure S1B, Table S10).

Linear mixed-effects modeling revealed a trend toward a longitudinal effect of HBOT sessions on CD27⁻CD4⁺ T-cell proportions (% of CD4⁺ T cells), although the overall time effect did not reach statistical significance (Time effect: F = 2.407, *p* = 0.068) (Table 3). Compared with baseline, CD27⁻CD4⁺ T-cell proportions were significantly reduced after 10 sessions (estimate = −1.089 percentage points, *p* = 0.040, 95% CI [−2.127, −0.050], Cohen’s d = −0.25) and 20 sessions (estimate = −1.118 percentage points, *p* = 0.036, 95% CI [−2.164, −0.073], Cohen’s d = −0.26). After further adjustment for age, sex, and BMI, the temporal trend remained similar and still did not reach statistical significance (F = 2.456, *p* = 0.063) (Additional file 1: Table S4).

Further interaction analyses indicated that demographic characteristics modified the longitudinal trajectory of CD27⁻CD4⁺ T-cell changes. A borderline interaction between treatment session and age was observed (Time × Age: F = 1.917, *p* = 0.078) (Table 4). Pairwise subgroup comparisons showed that the middle-aged group (40–59 years) exhibited lower CD27⁻CD4⁺ T-cell proportions compared with the elderly group (60–79 years) after 20 sessions (estimate = −2.812 percentage points, *p* = 0.013, 95% CI [−5.018, −0.607]). Age-stratified analysis further demonstrated that the reduction was most pronounced among middle-aged participants, with significant decreases after 20 sessions (estimate = −2.714 percentage points, *p* = 0.001), whereas elderly participants showed no significant longitudinal changes (Table 5).

Sex-stratified analysis demonstrated that females exhibited significant reductions after 10 sessions (estimate = −1.846 percentage points, *p* = 0.008) and 20 sessions (estimate = −1.434 percentage points, *p* = 0.040), whereas no significant changes were observed among males.

#### Changes in CD28⁻CD4⁺ T-cell proportions during HBOT

The proportion of CD28⁻CD4⁺ T cells remained relatively stable during HBOT treatment. Based on flow cytometric measurements, the mean proportion of CD28⁻CD4⁺ T cells was 10.20 ± 1.16% at baseline, 9.43 ± 1.06% after 10 sessions, 9.44 ± 1.07% after 20 sessions, and 9.65 ± 1.17% after 30 sessions (Figure S1B, Table S11).

Consistent with the relatively stable proportions observed, linear mixed-effects modeling did not demonstrate a significant overall effect of HBOT sessions on CD28⁻CD4⁺ T-cell proportions (% of CD4⁺ T cells), indicating limited longitudinal modulation of this senescence-associated CD4⁺ T-cell phenotype during the treatment course (Table 3).

Although the overall time effect was not significant, subgroup analyses suggested potential demographic-dependent responses. A borderline Time × Sex interaction was observed for CD28⁻CD4⁺ T-cell proportions (F = 2.248, *p* = 0.083) (Table 4). Further subgroup comparison showed that males exhibited less reduction than females after 20 sessions (estimate = 2.751 percentage points, *p* = 0.011, 95% CI [0.631, 4.870]). Consistently, sex-stratified analysis demonstrated that females showed a significant reduction after 20 sessions (estimate = −1.610 percentage points, *p* = 0.023), whereas males showed no significant changes throughout the treatment period (Table 5).

A similar trend was observed for BMI-related differences. The Time × BMI interaction showed a borderline effect (F = 2.248, *p* = 0.083) (Table 4). At 20 sessions, participants with normal weight (18.5 ≤ BMI < 24) exhibited lower CD28⁻CD4⁺ T-cell proportions compared with overweight participants (24 ≤ BMI < 28) (estimate = −2.809 percentage points, *p* = 0.010, 95% CI [−4.947, −0.672]). BMI-stratified analysis further demonstrated a significant reduction after 20 sessions in the normal BMI group (estimate = −1.543 percentage points, *p* = 0.027), whereas no significant modulation was observed in the overweight group (Table 5).

Overall, CD28⁻CD4⁺ T cells showed limited longitudinal changes following HBOT, with only selected subgroups (female participants and individuals with normal weight) demonstrating modest reductions, suggesting a weaker and more context-dependent response compared with CD8⁺ senescence-associated T-cell subsets.

### Sex- and age-related differences in plasma cytokine changes during short-term HBOT

#### Sex- and age-dependent modulation of plasma TNF-α concentrations during HBOT

The mean plasma TNF-α concentration showed a transient decline during the early phase of HBOT, decreasing from 214.84 ± 43.25 pg/mL at baseline to 141.35 ± 21.02 pg/mL after 10 sessions, followed by partial recovery at 20 sessions (209.45 ± 48.94 pg/mL) and 30 sessions (197.35 ± 49.80 pg/mL) (Figure S3, Table S12).

Although linear mixed-effects models indicated no significant overall time effect of HBOT on inflammatory cytokine levels, significant moderating effects of sex and age were observed at the 10-session timepoint. For tumor necrosis factor-alpha (TNF-α), the Time × Sex interaction at 10 sessions was significant, revealing a greater TNF-α reduction in males compared to females (Estimate = −202.85, *p* = 0.008). Stratified analyses of sex revealed this pattern: in males, TNF-α levels significantly decreased at 10 sessions (Estimate = −185.12 ± 59.97, *p* = 0.004), while the change in females was nonsignificant (Additional file 1: Table S5). Additionally, a marginally significant Time × Age interaction was observed at the 10 sessions (Estimate = −162.30, *p* = 0.053), suggesting that the middle-aged group may exhibit a more pronounced TNF-α reduction compared to elderly participants. Age-stratified analysis revealed that in the middle-aged group, TNF-α levels showed a significant decrease at 10 sessions (Estimate = −161.64 ± 56.10, *p* = 0.006) (Additional file 1: Table S6).

Together, these findings suggest that HBOT-associated TNF-α modulation was primarily observed during the early treatment phase and was influenced by participant characteristics, particularly sex and age.

#### Sex- and age-dependent modulation of plasma IL-1β concentrations during HBOT

The mean plasma IL-1β concentration showed a modest decrease during the early phase of HBOT, decreasing from 7.12 ± 0.99 pg/mL at baseline to 6.36 ± 0.59 pg/mL after 10 sessions, followed by a transient increase at 20 sessions (7.29 ± 1.00 pg/mL) and a subsequent decrease after 30 sessions (6.70 ± 0.81 pg/mL) (Figure S3, Table S13).

Linear mixed-effects modeling did not reveal a significant overall time effect on IL-1β concentrations. However, interaction analyses suggested demographic-dependent responses. A significant Time × Sex interaction was observed at 10 sessions, indicating greater IL-1β reduction in males than females (Estimate = −4.22, *p* = 0.015). Sex-stratified analyses revealed that in males, IL-1β levels significantly decreased at 10 sessions (Estimate = −3.58 ± 1.33, *p* = 0.011), while the change in females was non-significant (Additional file 1: Table S5). Additionally, a marginally significant Time × Age interaction was detected at 10 sessions (Estimate = −3.52, *p* = 0.052), suggesting that the middle-aged group may exhibit a more pronounced IL-1β reduction compared to elderly participants. Age-stratified analysis revealed that in the middle-aged group, IL-1β levels showed a significant decrease at 10 sessions (Estimate = −2.97 ± 1.17, *p* = 0.015) (Additional file 1: Table S6).

These results indicate that IL-1β responses to HBOT were characterized by an early, subgroup-specific reduction rather than a generalized decrease across the cohort.

#### Sex- and age-dependent modulation of plasma IL-10 concentrations during HBOT

The mean plasma IL-10 concentration showed a fluctuating pattern during HBOT treatment. Compared with baseline (8.23 ± 1.19 pg/mL), IL-10 levels slightly decreased after 10 sessions (7.59 ± 0.80 pg/mL), returned to baseline levels after 20 sessions (8.23 ± 1.22 pg/mL), and increased after 30 sessions (9.05 ± 1.26 pg/mL) (Figure S3, Table S14).

Linear mixed-effects modeling did not demonstrate a significant overall time effect on IL-10 concentrations. However, the Time × Sex interaction revealed that at 10 sessions, males had a greater IL-10 reduction than females (Estimate = −3.90, *p* = 0.034). Stratified analyses revealed that in males, IL-10 levels significantly decreased at 10 sessions (Estimate = −3.14 ± 1.46, *p* = 0.037), while the change in females was non-significant (Additional file 1: Table S5). Although a non-significant Time × Age interaction was detected in IL-10, age-stratified analyses revealed that in the middle-aged group, IL-10 levels showed a significant decrease at 10 sessions (Estimate = −2.58 ± 1.25, *p* = 0.046) (Additional file 1: Table S6).

## Discussion

An increase in relative telomere length was one of the most consistent longitudinal biomarker changes observed during the course of HBOT. The significant time effect across the post-baseline time points suggests that PBMC relative telomere length may increase during the early phase of HBOT exposure and remain elevated over the treatment course. This finding is broadly consistent with previous reports suggesting that intermittent hyperoxic exposure may influence telomere dynamics in peripheral blood cells [19, 25]. However, because the present study did not include a parallel non-HBOT control group or direct molecular measurements of telomerase activity, oxidative stress, DNA damage responses, or hypoxia-related signaling, the mechanisms underlying the observed telomere changes remain speculative.

Several mechanisms may be relevant to the observed changes in PBMC relative telomere length. Intermittent hyperoxic exposure during HBOT can generate fluctuations in oxidative and redox signaling, which may activate adaptive antioxidant pathways, including SOD- and Nrf2-related responses, and thereby reduce oxidative injury to telomeric DNA [52, 60]. HBOT may also engage hypoxia-responsive signaling through the so-called “hyperoxic-hypoxic paradox”. In this context, HIF-1α-related pathways have been linked to telomerase regulation and cellular repair processes, although these mechanisms were not directly measured in the present study [42, 45]. In addition, HBOT has been reported to mobilize stem and progenitor cells through VEGF and SDF-1 signaling, which may alter the composition of circulating immune cells and indirectly affect telomere-related measurements [53]. These coordinated responses support the hypothesis that HBOT facilitates telomere maintenance not by exogenous activation, but by enhancing the fidelity of intrinsic repair networks involved in redox balance, telomerase regulation, and stem cell mobilization.

An alternative explanation should be considered. The increase in PBMC relative telomere length may also be influenced by changes in the cellular composition of PBMCs. PBMC telomere length is an averaged measure across mixed immune cell populations, rather than a direct measure of telomere elongation within a specific cell type. Previous studies have shown that leukocyte telomere length can be affected by blood cell composition and that telomere length differs among lymphocyte subsets [31]. CD8⁺CD28⁻ T cells, in particular, have been reported to have shorter telomeres than CD28⁺ T cells [3, 33]. In this context, a reduction in short-telomere, senescence-associated T-cell subsets could increase the average telomere signal measured in bulk PBMCs, even without uniform telomere elongation in individual cells. This interpretation is compatible with the observed decreases in selected CD8⁺ senescence-associated T-cell subsets in our cohort. Studies using sorted immune cell subsets, single-cell telomere assays, telomerase activity measurements, and markers of apoptosis or cell turnover will be needed to clarify whether the observed change reflects true telomere elongation, immune cell compositional shifts, or both.

CD27 and CD28 are key co-stimulatory receptors involved in T-cell activation, differentiation, survival, and memory formation [1, 26, 56]. CD28 interacts with CD80/CD86 on antigen-presenting cells and provides essential co-stimulatory signals for IL-2 production, clonal expansion, survival, metabolic activation, and effector differentiation. With repeated antigenic stimulation and aging, CD28 expression is progressively lost, particularly in CD8⁺ T cells, leading to the accumulation of late-differentiated CD28⁻ populations with reduced proliferative potential, shortened telomeres, altered effector function, and increased inflammatory activity [22, 54, 56]. CD27, a member of the TNF receptor superfamily that binds CD70, supports T-cell survival, memory generation, and maintenance of antigen-specific responses [4, 28]. CD27 expression is generally retained in naïve and central memory T cells but declines during differentiation toward effector memory and terminally differentiated T-cell states [1, 26]. Therefore, loss of CD27 and/or CD28 is commonly interpreted as a marker of advanced T-cell differentiation and reduced immune reserve.

In this context, the HBOT-associated reductions in CD28⁻CD8⁺ and CD27⁻CD8⁺ T-cell subsets observed in our study may indicate remodeling away from late-differentiated, senescence-associated T-cell phenotypes. The stronger response observed in CD8⁺ rather than CD4⁺ subsets is biologically plausible, as CD8⁺ T cells are known to undergo more pronounced age-related loss of CD28 expression and expansion of late-differentiated effector populations than CD4⁺ T cells [12, 16, 54]. The observed reduction in CD27⁻ subsets further suggests that HBOT may be associated with changes in T-cell differentiation status, particularly within the CD8⁺ compartment. However, because absolute cell counts, functional assays, and detailed memory subset profiling were not performed, these findings should be interpreted as proportional phenotypic changes rather than definitive evidence of restored T-cell function or reversal of T-cell senescence.

The subgroup analyses suggested that reductions in senescence-associated T-cell subsets were more evident during the early phase of HBOT among younger participants, females, and individuals with normal BMI. This host-dependent pattern is biologically plausible. Younger individuals generally retain greater naïve T-cell availability, broader T-cell receptor diversity, preserved thymic output, and greater immune remodeling capacity, whereas older individuals accumulate late-differentiated memory T cells and show reduced immune plasticity [22, 56]. Consequently, CD28⁻ and CD27⁻ T-cell subsets in younger individuals may remain more amenable to phenotypic remodeling, whereas older adults exhibit more fixed immunosenescence resulting from progressive immune remodeling [43], accompanied by impaired mitochondrial function and reduced metabolic flexibility [37], thereby limiting the early response to HBOT. Sex-related immune differences may also be relevant, as females often exhibit stronger innate and adaptive immune responses than males, partly influenced by sex chromosomes and estrogen-related immune regulation [30, 36]. Similarly, BMI may further modify immune responsiveness, because excess adiposity is associated with chronic low-grade inflammation, metabolic stress, and accelerated immunosenescence, all of which may promote the persistence of late-differentiated CD28⁻/CD27⁻ T cells and reduce their responsiveness to HBOT [14, 18]. Thus, younger age, female sex, and normal BMI may reflect a more responsive immune-metabolic state, allowing more evident early phenotypic remodeling of late-differentiated T-cell subsets during HBOT. In contrast, the less pronounced short-term responses observed in males, older participants, and overweight individuals do not necessarily indicate absence of biological responsiveness, but may reflect a slower remodeling process or a higher threshold for detectable changes. These populations may require longer exposure or a greater number of HBOT sessions before comparable reductions in senescence-associated T-cell subsets become evident. However, because these subgroup findings were exploratory and the study was not designed to compare different treatment durations across subgroups, this interpretation should be regarded as hypothesis-generating and requires validation in future controlled studies.

Although HBOT has been reported to suppress pro-inflammatory mediators such as IL-1β and TNF-α through modulation of NF-κB signaling and activation of antioxidant pathways including Nrf2 [2, 46], our findings indicate that short-term HBOT did not induce uniform cytokine changes across the entire cohort. Instead, subgroup analyses revealed more pronounced early reductions in IL-1β and TNF-α among males and middle-aged participants. This heterogeneity may reflect sex- and age-related differences in baseline inflammatory states, immune responsiveness, or cytokine regulatory networks, which are known to influence immune aging trajectories and inflammatory profiles [36]. Notably, the concurrent decrease in IL-10, a cytokine with well-established anti-inflammatory functions, may appear counterintuitive, as increased IL-10 levels have been reported following HBOT in some studies [50]. However, a similar reduction in IL-10 has also been observed in other contexts [49]. Although IL-10 is widely recognized as an anti-inflammatory cytokine, its circulating levels reflect a dynamic balance between inflammatory activation and regulatory feedback rather than a simple indicator of immune suppression or activation. IL-10 expression can vary according to inflammatory burden, immune context, and temporal stage of immune responses [48]. This pattern may be compatible with a transition from a state of heightened inflammatory activation and compensatory regulation toward a more balanced immune homeostatic state.

Taken together, our findings suggest that short-term HBOT induces coordinated but temporally distinct modulation of immune aging-related biomarkers, including telomere dynamics, senescent-like T-cell phenotypes, and inflammatory profiles. Importantly, these responses were not uniform across individuals but appeared to be influenced by biological factors including age, sex, and BMI. Such heterogeneity may reflect differences in immune reserve, inflammatory status, and cellular adaptability, resulting in distinct patterns of immune remodeling after HBOT. Nonetheless, this study has several limitations. First, although the prospective within-subject repeated-measures design reduced inter-individual variability, the absence of a parallel non-HBOT or sham-control group limits causal inference. Time-dependent confounding factors, including lifestyle changes, concurrent treatments, and regression to the mean, cannot be fully excluded; therefore, the observed biomarker changes should be interpreted as HBOT-associated changes rather than definitive causal effects. Second, the study population was clinically heterogeneous, with participants receiving HBOT for different underlying conditions that may have influenced baseline immune states and longitudinal biomarker trajectories. Third, these biomarkers captured distinct biological compartments, including PBMC telomere dynamics, T-cell phenotypes, and circulating cytokine profiles, and therefore may not reflect a unified regulatory mechanism. Fourth, T-cell senescence was assessed based on CD27 and CD28 expression without functional validation; additional functional and molecular assessments, including proliferative capacity, DNA damage responses, telomerase activity, and senescence-related pathways, are required to determine whether the observed changes represent true immune rejuvenation or transient phenotypic remodeling. Accordingly, these subsets should be interpreted as senescence-associated T-cell phenotypes rather than direct evidence of functional T-cell senescence. Finally, cytokine measurements were limited to selected plasma markers and showed substantial inter-individual variability, and subgroup analyses by age, sex, and BMI were exploratory and not powered for definitive subgroup-specific conclusions. Future randomized controlled studies with larger sample sizes, more homogeneous cohorts, predefined subgroup analyses, and integrated functional immune profiling are warranted to validate and extend these findings.

## Conclusion

In summary, this prospective observational study suggests that short-term 2.5 ATA HBOT is associated with coordinated changes across multiple senescence-related domains, including increased relative telomere length in PBMCs, reduced proportions of senescence-associated T-cell subsets, and dynamic modulation of circulating inflammatory profiles. Importantly, these responses were not uniform across individuals; subgroup analyses revealed that age, sex, and BMI were associated with distinct patterns of HBOT-related immune remodeling, suggesting potential differences in individual responsiveness. These findings support HBOT as a potential strategy for modulating immune aging and highlight the importance of personalized approaches in future applications. Further randomized controlled studies with longer follow-up and functional immune assessments are warranted to determine the durability, underlying mechanisms, and clinical relevance of HBOT-induced immune remodeling.

## Supplementary Information

Supplementary Material 1.

## Data Availability

Data generated and/or analyzed during the current study are available from the corresponding author on reasonable request.

## Abbreviationa

AIC: Akaike Information Criterion
BIC: Bayesian Information Criterion
CRP: C-reactive protein
HBOT: Hyperbaric oxygen therapy
LMM: Linear mixed-effects model
MFI: Median fluorescence intensity
PBMCs: Peripheral blood mononuclear cells
REML: Restricted maximum likelihood
SASP: Senescence-associated secretory phenotype
VEGF: Vascular endothelial growth factor
WGOC: Working Group on Obesity in China
